# The prevalence of adverse childhood experiences in systematic reviews of primary headache and cancer

**DOI:** 10.1192/j.eurpsy.2025.1488

**Published:** 2025-08-26

**Authors:** A. Mavromanoli, C. Sikorski, D. Behzad, K. Manji, C. Kreatsoulas

**Affiliations:** 1 Johannes Gutenberg University, Mainz, Germany; 2 McMaster University, Hamilton; 3 Brock University, St. Catharines; 4 University of Toronto, Toronto, Canada; 5 Harvard T.H. Chan School of Public Health, Boston, United States

## Abstract

**Introduction:**

Adverse childhood experiences (ACEs) describe an array of stressful exposures that occur during childhood/ adolescence, including types of abuse and neglect. As ACEs occur during a critical period of human development, they have been associated with a range of chronic diseases in adulthood.

**Objectives:**

We aimed to systematically analyze the prevalence of ACEs in studies in two different disease severity populations, including primary headache and cancer. The objectives of this study are to examine,

The prevalence of ACEs hypothesized to impact chronic disease

The pooled association of the presence of “disease”, which consists for this study only primary headache and/or cancer, with ACEs

**Methods:**

The inclusion criteria for the studies in the systematic reviews/meta-analyses included observational studies with a comparator group, ACE(s) occurrence at ≤18 years of age, and disease diagnosis at ≥21 years of age. Searches were conducted up to Mar 16, 2023. Two review authors independently screened articles, extracted data, assessed risk of bias using QUIPS, and conducted GRADE assessment as part of the systematic reviews. In this study, we calculated the prevalence and the odds of experiencing ACEs among those with disease compared to those without disease.

**Results:**

Of the total 39,658 articles screened, 44 studies were eligible for synthesis of 437,852 participants from 22 countries across five continents. Among included studies, the most commonly examined ACEs were physical abuse (82% of studies), sexual abuse (77%), household substance or alcohol abuse (52%), witnessing or threat of violence (50%), having a household member with a mental illness (45%), and emotional abuse (43%).

Of the 437,852 participants in the synthesis, an estimated total of 196,587 participants (45%) reported experiencing at least one ACE. Among participants with primary headache and/or cancer, 51% (29,838/58,580) reported experiencing at least one ACE, compared to 44% (166,749/379,272) of participants without primary headache and/or cancer (crude odds ratio = 1.32, 95% confidence interval: 1.30, 1.35).

**Conclusions:**

The prevalence of ACEs is high when hypothesized to be associated with diseases, independent of the risk for mortality (i.e., primary headache low mortality, cancer high mortality). Abuse ACEs are more commonly considered in studies than neglect ACEs, and therefore, more consistent and comprehensive reporting of ACEs is needed. There is untapped opportunity for the expertise of psychiatrists to collaborate with clinicians and researchers of adult chronic disease studies.

**Disclosure of Interest:**

None Declared

